# Magnetic Properties of Fe-Ni-Cr Alloy Coatings Processed via High-Velocity Oxygen-Fuel Spraying

**DOI:** 10.3390/ma19194258

**Published:** 2026-10-08

**Authors:** Xiqiang Ma, Shuai Han, Dongliang Jin, Jinpeng Li, Nan Guo

**Affiliations:** 1 School of Mechatronics Engineering, Henan University of Science and Technology, Luoyang 471000, China; maxiqiang@haust.edu.cn (X.M.); 18239575731@163.com (S.H.); ziranheping@163.com (D.J.); 2 Xinchang Technology Achievement Transformation Center, Henan University of Science and Technology, Shaoxing 312500, China; ghost2233@163.com; 3 Zhejiang Sling Intelligent Drive Group Co., Ltd., Shaoxing 312500, China

**Keywords:** high-velocity oxygen-fuel (HVOF) spraying, Fe-Ni-Cr alloy, soft magnetic coatings, background signal stripping, magnetic properties

## Abstract

**Highlights:**

**What are the main findings?**

- HVOF preserves the FCC lattice of Fe-Ni-Cr powder without massive oxidation.
- Intrinsic magnetic data reveal a unique wasp-waisted distortion in low fields.
- Thinner coatings show lower coercivity, as thicker ones accumulate residual stress.

**What are the implications of the main findings?**

- A signal stripping method effectively extracts true magnetic data of thin coatings.
- Dual-phase asynchronous magnetization explains the wasp-waisted distortion.
- Fe-Ni-Cr coatings offer a viable solution for in-situ bearing condition monitoring.

**Abstract:**

In this work, soft magnetic coatings were fabricated from Fe-Ni-Cr (1J38) alloy powders via high-velocity oxygen-fuel (HVOF) spraying. The microstructural features and intrinsic magnetic properties were characterized using scanning electron microscopy (SEM), X-ray diffraction (XRD), and a background signal stripping algorithm. Furthermore, the microstructural defects and magnetic degradation characteristics of coatings with varying thicknesses were comparatively analyzed. The results indicate that while the HVOF deposits exhibit exceptional density, the rapid quenching process inevitably induces oxygen-rich inter-splat boundary networks and residual stresses within the coatings. XRD analysis reveals no significant macroscopic oxidation or phase transformation during deposition, demonstrating a high-fidelity retention of the original face-centered cubic (FCC) lattice structure of the feedstock alloy. Magnetic measurements, upon the precise elimination of substrate background interference, reveal a distinct wasp-waisted magnetization distortion at low magnetic fields. This phenomenon can be attributed to the asynchronous magnetization response between the easily reversed, unoxidized soft-magnetic splat cores and the high-barrier boundary pinning networks (composed of inter-splat oxide interlayers and localized high-stress concentrations) within the coating. Compared to the thicker deposits (0.5 mm), the thinner coatings (0.3 mm) exhibit superior magnetic performance, characterized by a lower intrinsic coercivity. This degradation in thicker coatings is ascribed to the substantial accumulation of macroscopic residual stresses during deposition, which exert a stronger pinning effect on magnetic domain wall motion.

## 1. Introduction

Soft magnetic materials, serving as the essential media for the conversion and transmission of magneto-electric signals, play an indispensable fundamental role in modern electromagnetic compatibility (EMC), high-precision magnetic sensors, and microelectronic components [1,2,3]. Among various soft magnetic systems, Fe-Ni-Cr alloys (e.g., 1J38 Permalloy) have garnered profound interest from both academia and industry owing to their exceptionally high initial permeability, ultra-low coercivity (*H*_c_), and the superior corrosion resistance alongside high electrical resistivity at high frequencies imparted by chromium (Cr) alloying [4,5]. However, traditional bulk soft magnetic alloys possess significant inherent limitations: they are acutely sensitive to mechanical stresses, and conventional subtractive machining inevitably induces lattice distortions and macroscopic residual stresses, which severely deteriorate intrinsic magnetic properties via the strong pinning of magnetic domain walls; furthermore, their relatively high density restricts their potential for lightweight applications in sectors such as aerospace. Nevertheless, driven by their excellent comprehensive magnetic properties, their potential application boundaries are continuously expanding, exhibiting tremendous engineering potential particularly in miniaturized high-frequency inductors, non-destructive testing (NDT), and in-situ early-warning sensors for the thermal-induced failure of critical bearings in high-end equipment based on the magneto-thermal coupling effect. Currently, as modern electronic devices evolve towards lightweighting, miniaturization, and conformal integration with complex curved surfaces, traditional bulk soft magnetic materials face severe physical bottlenecks in terms of forming and weight control [6]. Therefore, employing surface engineering technologies to deposit high-performance soft magnetic coatings onto non-magnetic or structural components, thereby achieving surface magnetic functionalization, has emerged as a significant research frontier in contemporary materials science [7,8,9].

Particularly within the realm of intelligent monitoring for high-end equipment, the magnetic functionalization imparted by soft magnetic coatings demonstrates indispensable application value. For instance, in bearing temperature monitoring, traditional contact-based thermometry compromises structural integrity, whereas existing non-contact techniques are highly susceptible to severe interference from enclosed spaces and complex oil-gas lubrication environments. To overcome this bottleneck in thermal-induced failure monitoring, recent studies have proposed depositing Fe-Ni-Cr soft magnetic alloy films (e.g., 1J38) onto bearing inner rings, which, coupled with eddy current sensors, enables the in-situ and non-destructive temperature monitoring of critical moving components [9]. The core of this cutting-edge technology fundamentally relies on the highly sensitive permeability-temperature coupling characteristics of the 1J38 alloy under alternating magnetic fields. Consequently, achieving the high-quality deposition of these alloys as coatings onto complex structural components, alongside precisely elucidating their as-deposited intrinsic magnetic properties, has emerged as a critical prerequisite for advancing such advanced intelligent sensor systems toward engineering applications.

Among various coating deposition technologies, high-velocity oxygen-fuel (HVOF) spraying is regarded as an ideal approach for depositing high-quality metallic magnetic coatings owing to its distinct process characteristics of high kinetic energy and low thermal input [10,11]. Compared to conventional plasma spraying, HVOF can significantly suppress the in-flight high-temperature oxidation of alloy powders, maximally preserving their intrinsic phase structures and thereby yielding exceptionally dense coatings [12]. However, HVOF deposition is inherently an extreme non-equilibrium thermodynamic process. Following supersonic impact and ultra-fast quenching, the powder particles inevitably undergo complex microstructural evolutions within the coating, encompassing a typical lamellar splat morphology, severe lattice distortions, microscopic residual porosity, and substantial internal residual stress gradients [13]. These process-induced, heterogeneous microstructural defect networks are bound to exert a profound influence on the domain wall motion and macroscopic magnetization dynamics of the material [14,15].

Although the application of HVOF technology in wear-resistant and anti-corrosion fields has matured, mechanistic research regarding its application in soft magnetic coatings still exhibits significant knowledge gaps and faces two primary critical challenges. First, the mechanisms by which heterogeneous microstructural defects govern magnetic domain reversal remain elusive. The slight surface oxidation of molten droplets during their in-flight transit through the high-temperature flame jet evolves into an intricate network of oxygen-rich inter-splat boundaries upon coating densification. This oxide network, coupled with the high-stress regions induced by severe plastic deformation, exerts a strong pinning effect on domain wall motion; however, a systematic demonstration of how it specifically dictates the deterioration of the macroscopic magnetic hysteresis loop is still lacking [16,17,18]. Second, significant measurement inaccuracies frequently occur during the evaluation of the coatings’ magnetic properties. When characterizing ultra-thin soft magnetic coatings via vibrating sample magnetometry (VSM), the total magnetic response of the composite sample is exceptionally susceptible to severe interference from substrate background signals—manifesting either as an artificially inflated saturation magnetization caused by a ferromagnetic substrate or as a distorted low-field signal-to-noise ratio induced by trace ferromagnetic impurities within a diamagnetic substrate (e.g., copper). The existing literature frequently overlooks this substrate coupling effect, and there is a conspicuous scarcity of systematic methodological investigations into accurately stripping the substrate interference to extract the intrinsic magnetic properties of the coatings, which has consequently led to widespread data misinterpretation and mechanistic fallacies.

To address the aforementioned research challenges, this study systematically investigates the intrinsic correlation between the microstructural evolution and the intrinsic magnetic properties of 1J38 soft magnetic alloy coatings fabricated via the HVOF process. To circumvent the signal-to-noise ratio distortion caused by substrate background fluctuations during the measurement of ultra-thin coatings, coatings with two distinct thicknesses (0.3 mm and 0.5 mm) were deposited onto three typical substrates exhibiting significant thermophysical and magnetic disparities (42CrMo ferromagnetic steel, Cu plates, and GCr15 bearing steel), thereby yielding a total of six representative experimental sample groups. A rigorous background signal stripping algorithm [19] was introduced to successfully eliminate the comprehensive background errors originating from the substrates and the testing environment, thereby accurately reconstructing the intrinsic magnetic hysteresis loops of the coatings. Furthermore, by coupling physical property measurement system (PPMS) characterizations of the feedstock powders with cross-sectional SEM/EDS analyses, this study provides an in-depth elucidation of the unique wasp-waisted magnetization signature observed in the low-field region [20], proposing a dual-phase asynchronous magnetization competition model comprising soft-magnetic splat cores (unoxidized 1J38 phases) and high-barrier boundary pinning networks (oxygen-rich boundaries and localized high-stress concentrations). This research not only elucidates the underlying physical mechanisms governing the macroscopic magnetic degradation induced by the evolution of coating thickness, but also establishes a crucial methodological reference for the high-precision magnetic characterization of thin soft magnetic coatings.

## 2. Materials and Methods

### 2.1. Materials Preparation

The spraying feedstock utilized in this study was a commercial gas-atomized 1J38 soft magnetic alloy powder supplied by Beijing Yanbang New Materials Technology Co., Ltd. (Beijing, China). As detailed in Table 1, its chemical composition consists of 37.82% Ni, 12.92% Cr, 0.46% Mn, 0.25% Si, trace amounts of C (0.04%), S (0.02%), and P (0.02%), with the balance being Fe. The specified particle size range of the product is 15–53 μm. Fabricated via a gas atomization process, the powder particles exhibit a highly spherical morphology. The measured particle size distribution reveals D10 = 18.33 μm, D50 = 34.97 μm, and D90 = 56.73 μm. Regarding fundamental physical properties, the powder demonstrates a flowability of 13.86 s/50 g, an apparent density of 4.31 g/cm^3^, and a tap density of 5.28 g/cm^3^. These characteristics optimally satisfy the stringent requirements for continuous and uniform powder feeding during the high-velocity oxygen-fuel (HVOF) spraying process. Notably, the solid-solution alloying of Cr not only enhances the high-frequency electrical resistivity of the alloy to mitigate eddy current losses but also endows the resulting coatings with excellent corrosion resistance.

To systematically investigate the influence of substrate thermal conductivity and background magnetic signals on the evaluation of coating performance, three types of substrate materials exhibiting typical compositional and physical disparities were selected: (1) 42CrMo medium-carbon alloy steel (moderate thermal conductivity, typical ferromagnetism); (2) Cu substrates (exceptionally high thermal conductivity, macroscopic diamagnetism with only trace ferromagnetic impurities in the background); and (3) GCr15 bearing steel (high hardness, strong ferromagnetism). Prior to spraying, all substrate specimens were machined into standardized rectangular blocks (40 mm × 40 mm × 3 mm). The substrate surfaces were then mechanically polished using abrasive paper to remove native oxide layers and machining defects, thereby enhancing surface homogeneity. Subsequently, the substrates were ultrasonically cleaned sequentially in anhydrous ethanol and acetone to eliminate residual oils and impurities, ensuring effective contact between the molten particles and the substrates during the deposition process. Nonmagnetic refractory substrates (e.g., quartz, ceramics) were not employed because their inherent brittleness leads to severe cracking during grit-blasting and supersonic particle impingement, while their low thermal conductivity would fundamentally alter splat quenching kinetics. Consequently, high-purity Cu plates-combining metallic impact ductility, rapid heat dissipation, and macroscopic diamagnetism (*M*_s_ ≈ 0 emu/g)-serve as the optimal model substrate for intrinsic magnetic characterization.

Furthermore, following the coating deposition, the composite samples were precisely sectioned into miniature specimens (e.g., 3 mm × 3 mm × 3 mm blocks for VSM measurements) to accommodate the dimensional constraints of the equipment used for subsequent vibrating sample magnetometry and cross-sectional microstructural characterizations. The sectioning was performed utilizing low-speed wire electrical discharge machining continuously flooded with coolant. This method was specifically adopted to strictly preclude any secondary interference with the intrinsic magnetic properties of the materials arising from machining-induced thermal effects or additional deformation. The typical chemical compositions and macroscopic reference magnetic parameters of the three substrate materials are summarized in Table 2.

### 2.2. Coating Deposition

The 1J38 soft magnetic coatings were deposited onto the three aforementioned substrates utilizing a commercial HVOF spraying system. To further enhance the mechanical interlocking between the coating and the substrate and thereby improve adhesion strength, the substrate surfaces were subjected to a grit-blasting roughening treatment using 160-mesh white alumina abrasives prior to deposition. This grit-blasting process significantly increases the surface roughness of the substrate, providing abundant mechanical anchoring sites for the molten powder particles, which in turn ensures deposition stability. Following grit-blasting, the substrates were rigorously re-cleaned and dried to preclude any residual abrasives or contaminants from compromising the microstructural integrity and properties of the resulting coatings. During the HVOF process, propane was employed as the fuel gas and high-pressure oxygen as the oxidizing agent. The in-flight oxidation of the powder particles was minimized to the greatest extent by meticulously optimizing the spray distance and powder feed rate. The detailed spraying process parameters are summarized in Table 3.

To investigate the accumulation of residual stresses and the thermal effects induced by varying deposition thicknesses, coatings with target thicknesses of 0.3 mm and 0.5 mm were deposited onto each substrate by precisely controlling the number of reciprocating passes of the spray gun. Consequently, a core experimental matrix comprising six distinct groups (3 substrate types × 2 coating thicknesses) was established, as detailed in Table 4.

### 2.3. Microstructural and Phase Characterization

The phase compositions of both the substrates and the as-deposited coating surfaces were analyzed using an X-ray diffractometer (XRD, Bruker D8 Advance, Karlsruhe, Germany) equipped with Cu Kα radiation. The scanning was performed over a 2*θ* range of 10° to 90° at a scan rate of 5°/min. High-resolution observations of the powder surface morphology, the cross-sectional microstructures of the coatings, and the coating/substrate bonding interfaces were conducted utilizing a field-emission scanning electron microscope (FE-SEM, ZEISS Sigma 300, Oberkochen, Germany) equipped with an energy-dispersive X-ray spectroscopy (EDS) system. Furthermore, elemental mapping techniques were employed to specifically trace the distribution characteristics of oxygen within the coating cross-sections, aiming to elucidate the formation of oxygen-rich inter-splat boundary networks.

### 2.4. Magnetic Characterization and Substrate Signal Stripping

Initially, the bulk hysteresis loop of the raw 1J38 powder was measured at room temperature utilizing a Physical Property Measurement System (PPMS, Quantum Design DynaCool-14, San Diego, CA, USA) equipped with a VSM option. Because the pristine gas-atomized powder possesses an ultra-low coercivity (*H*_c_ ≈ 1.6 Oe), the PPMS was specifically chosen for its highly homogeneous superconducting magnetic field and exceptional low-field sensitivity, thereby establishing an absolute, artifact-free baseline for the material prior to any thermal or mechanical deformation. Subsequently, a standalone Vibrating Sample Magnetometer (VSM, LakeShore 8604, Westerville, OH, USA) was employed to characterize the macroscopic magnetic response of all six groups of rigid composite samples (coating + substrate, 3 mm × 3 mm × 3 mm) as well as their corresponding bare substrates under a maximum applied field of ±20,000 Oe at room temperature. The open air-gap architecture and rigid sample-positioning mechanism of the LakeShore VSM provide strictly reproducible spatial centering and geometry between the composite specimens and their bare references, which is indispensable for the high-precision paired background signal subtraction detailed below. Both systems share the same electromagnetic induction detection principle and were cross-calibrated using standard pure nickel references, ensuring full comparability across the datasets.

During VSM measurements, ultra-thin soft magnetic coatings are highly susceptible to severe interference from the substrate background. For coatings deposited on 42CrMo (Coat1, Coat4) and GCr15 (Coat3, Coat6) substrates, the overwhelming saturation magnetic moment of the substrates completely obscures the weak magnetic signals of the coatings, rendering the directly acquired data entirely inadequate for characterizing the intrinsic physical behavior of the coatings. Consequently, utilizing the relatively weakly magnetic Cu substrates (Coat2, Coat5) for signal decoupling emerges as the only viable approach to extract the intrinsic magnetic properties of the coatings. It should be emphasized that although Cu exhibits a higher coefficient of thermal expansion (*α*_Cu_ ≈ 16.8 × 10^−6^ K^−1^) than structural steels (*α*_steel_ ≈ 11.8~12.2 × 10^−6^ K^−1^), Cu is adopted here primarily as an indispensable model substrate. This methodological choice enables the mathematical decoupling and baseline reconstruction of the deposit’s intrinsic magnetic signals, which are otherwise completely overwhelmed by the strong ferro-magnetic steel background. Nevertheless, even with high-purity Cu substrates, the integrated background—including contributions from the mounting tape and sample rod—often contains non-linear fluctuations originating from trace ferromagnetic impurities. To address this, independent background magnetization calibrations were performed on bare substrates of identical specifications (inclusive of the sample rod and accessories) under the exact same testing conditions. A direct background subtraction method was subsequently employed to accomplish signal decoupling. The intrinsic magnetization of the coating is thus determined by precisely deducting the background magnetic moment of the bare substrate from the total magnetic moment of the composite sample, followed by mass normalization. The mathematical stripping model is formulated as follows:

(1) $$M_{\mathrm{coat}}(H)\;=\;\frac{m_{\mathrm{total}}(H)\;-\;m_{\mathrm{sub}}(H)}{\omega_{\mathrm{coat}}}$$

where *H* is the applied magnetic field strength, *M*_coat_(*H*) represents the intrinsic mass magnetization of the coating; *m*_total_(*H*) and *m*_sub_(*H*) are the total magnetic moment of the composite specimen and the background magnetic moment of the corresponding bare substrate measured by VSM, respectively; *ω*_coat_ denotes the net mass of the soft magnetic coating. This background subtraction treatment effectively eliminates the interference from substrate fluctuations, thereby restoring the true intrinsic low-field magnetic properties of the ultra-thin coatings.

## 3. Results

### 3.1. Morphology and Intrinsic Magnetic Properties of the Raw 1J38 Powder

**Figure 1:**
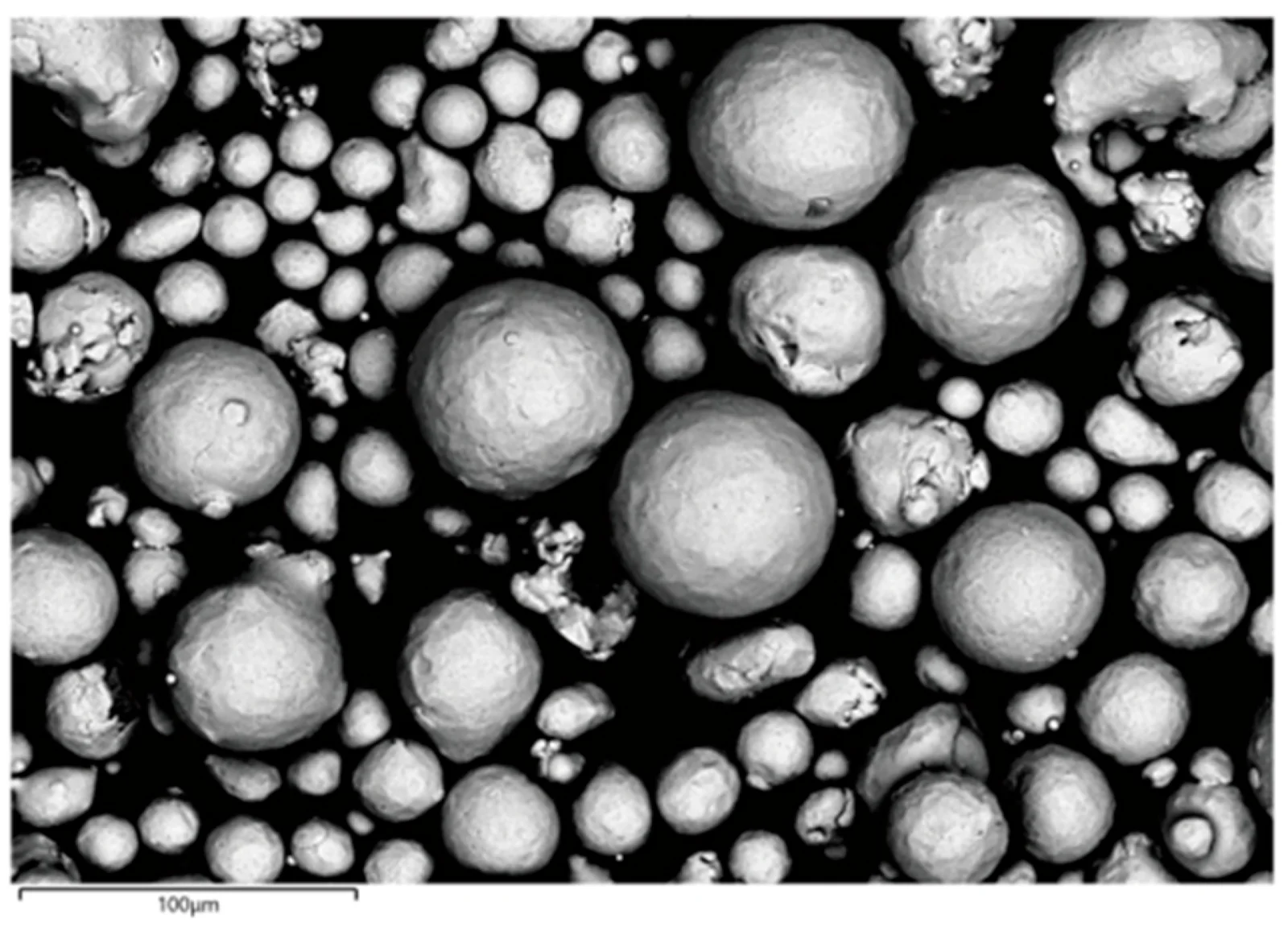
Surface morphology of the raw gas-atomized 1J38 soft magnetic alloy powder.

Figure 1 presents the surface morphological characteristics of the 1J38 soft magnetic alloy powder utilized in this study [21]. As clearly observed from the scanning electron microscopy (SEM) images, the powder exhibits a high degree of sphericity with a smooth, dense surface and is virtually devoid of attached satellite particles. Microscopically, the 2D visual field reveals fine spherical particles (15~25 μm) naturally nesting within the interstitial spaces among larger particles (35~55 μm). While this visual contrast in number frequency might superficially resemble two distinct populations, volumetric laser diffraction confirms that the powder possesses a continuous, unimodal distribution without statistically significant bimodal separation [22]. This natural interstitial packing of fine and coarse spherical particles effectively optimizes powder packing density, ensuring uniform heating, consistent acceleration, and stable deposition behavior within the high-velocity HVOF flame jet.

**Figure 2:**
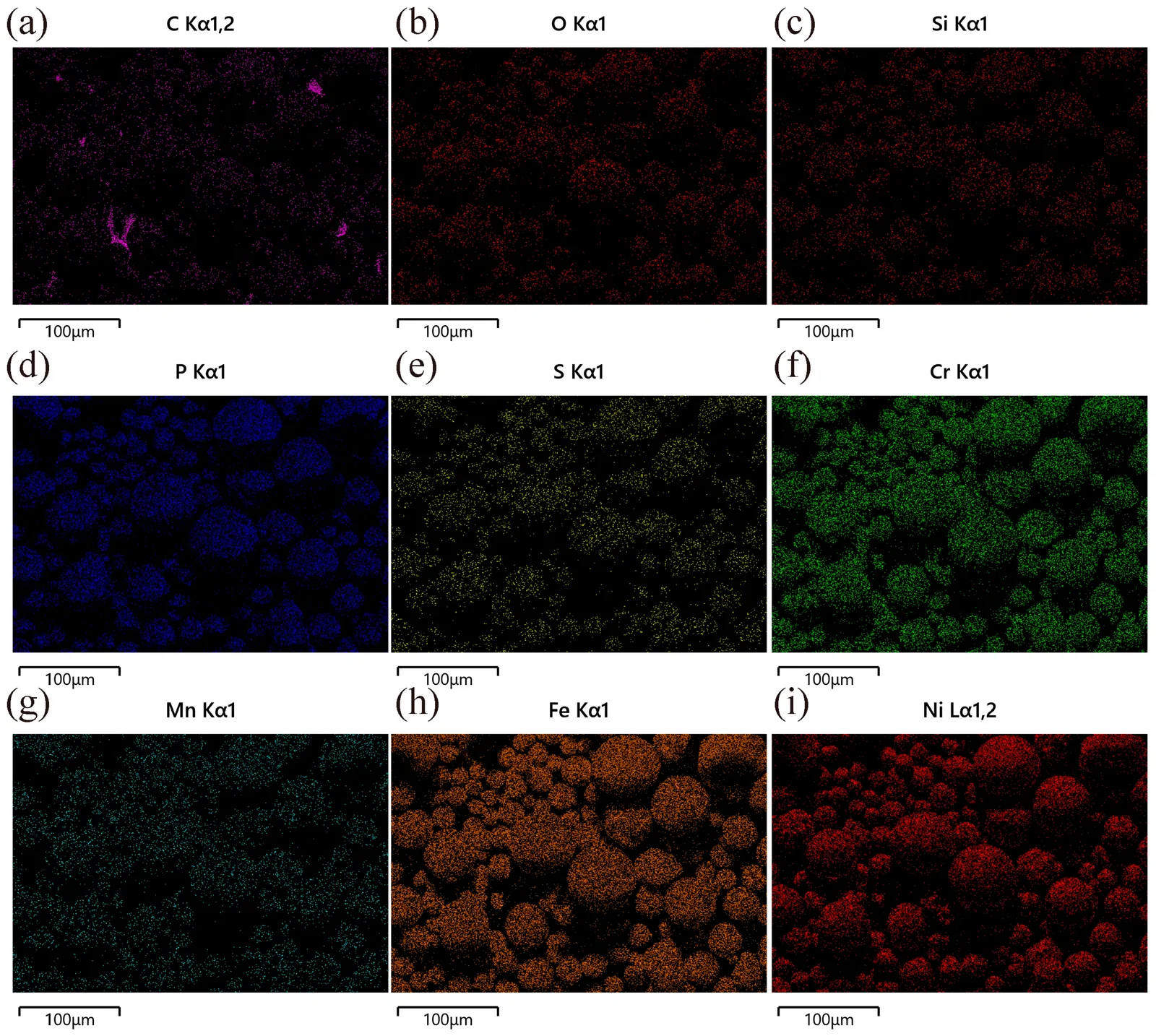
EDS elemental mapping of the raw 1J38 powder: (**a**) C Kα_1,2_, (**b**) O Kα_1_, (**c**) Si Kα_1_, (**d**) P Kα_1_, (**e**) S Kα_1_, (**f**) Cr Kα_1_, (**g**) Mn Kα_1_, (**h**) Fe Kα_1_, and (**i**) Ni Lα_1,2_. The maps reveal a homogeneous distribution of all major alloying elements (C, Si, P, S, Cr, Mn, Fe, Ni) throughout the powder particles. Notably, the oxygen map (**b**) exhibits only a faint, diffuse background signal, qualitatively indicating the absence of macroscopic oxide scales or surface oxide films on the as-received gas-atomized powder. Scale bars: 100 μm for all images.

To further investigate the elemental purity and spatial distribution within the powder, EDS elemental mapping analysis was conducted. As illustrated in Figure 2, the core magnetic elements (Fe, Ni) and the corrosion-resistant dopant (Cr) exhibit a highly uniform, long-range distribution across individual spherical particles, with no discernible elemental segregation or secondary phase agglomeration. Furthermore, qualitative EDS mapping reveals only a faint, discrete background oxygen signal without continuous oxide shells or localized segregation on the particle surfaces. This qualitative observation is consistent with the commercial gas-atomization manufacturing specification for 1J38 feedstock, which guarantees an ultra-low interstitial oxygen content (typically <0.03 wt.%) [23].

Benefiting from the aforementioned ideal microstructure, the bulk powder exhibited an excellent intrinsic soft magnetic response during PPMS measurements. As detailed in the subsequent comparative analysis (Section 3.4), the pristine 1J38 powder possesses an exceptionally low intrinsic coercivity (*H*_c_ ≈ 1.6 Oe) and a high saturation magnetization (*M*_s_ ≈ 28.44 emu/g). Notably, due to its near-perfect spherical morphology, the powder exhibits a substantial macroscopic demagnetizing factor (*N*_d_ ≈ 1/3). This induces a strong reverse demagnetizing field within the particles, significantly impeding the rotation of magnetic domains toward the applied external field [24,25]; consequently, the initial magnetization curve of the powder displays a pronounced sheared characteristic. 

### 3.2. Microstructural and Phase Stability Evaluation of the Coatings

**Figure 3:**
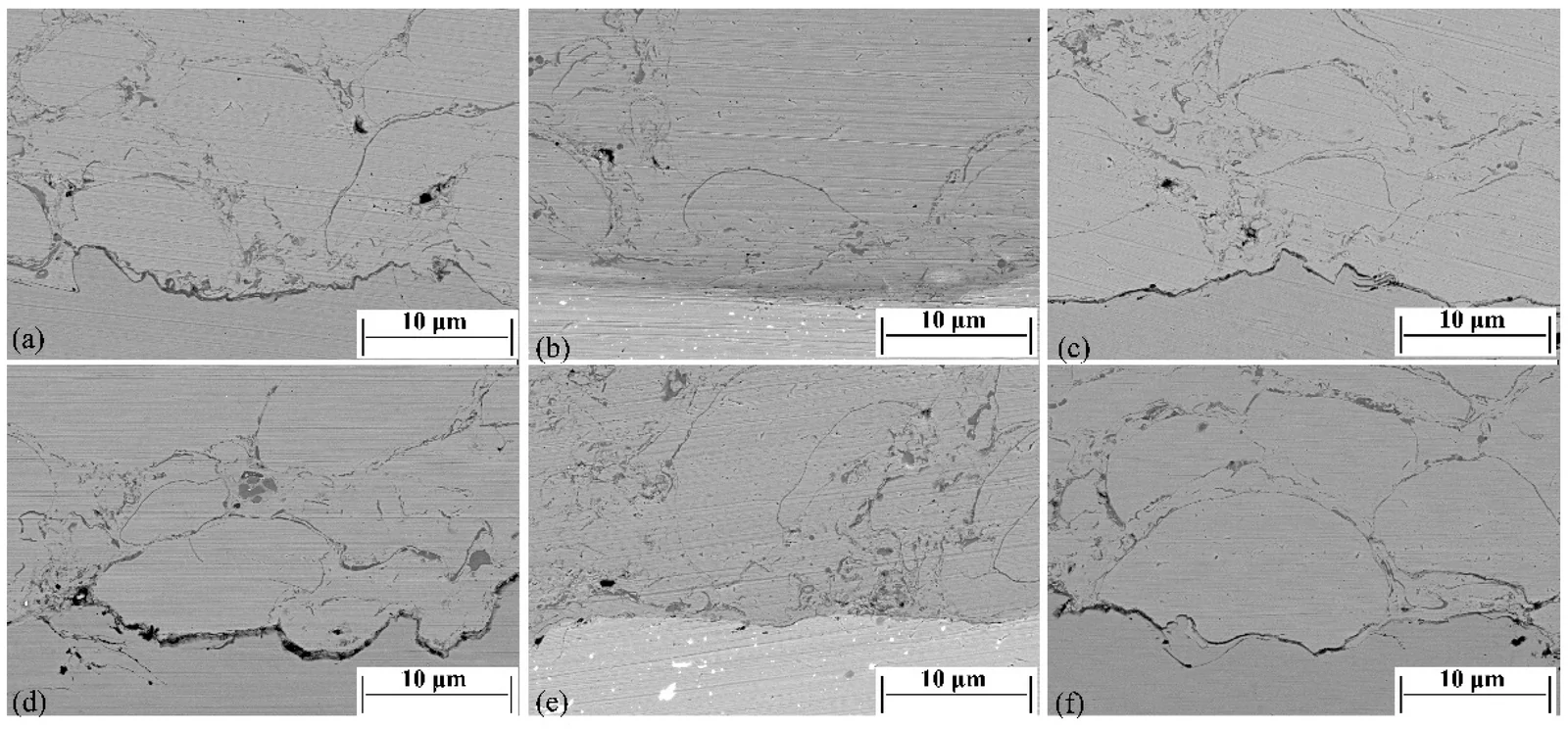
Cross-sectional SEM images of HVOF-sprayed 1J38 coatings: (**a**) Coat1 (42CrMo, 0.3 mm); (**b**) Coat2 (Cu, 0.3 mm); (**c**) Coat3 (GCr15, 0.3 mm); (**d**) Coat4 (42CrMo, 0.5 mm); (**e**) Coat5 (Cu, 0.5 mm); (**f**) Coat6 (GCr15, 0.5 mm). Scale bar: 10 μm.

Initially, cross-sectional morphological observations (Figure 3) reveal that the HVOF-sprayed 1J38 coatings exhibit exceptional overall compactness, with internal porosity being effectively maintained at an extremely low level [26]. No distinct microcracks or continuous delamination were observed at the bonding interfaces between the coatings and various substrates, demonstrating excellent mechanical interlocking and localized micro-metallurgical bonding characteristics [27]. Notably, the discontinuous dark layer observed along the steel interfaces (Figure 3a,c,d,f) originates from localized substrate oxidation and embedded Al_2_O_3_ grit residues, whereas Cu interfaces (Figure 3b,e) remain clean due to high thermal dissipation. Within the coatings, the characteristic lamellar structure composed of stacked, flattened splats, which is typical of thermal spraying processes, can be clearly identified. Although the HVOF process is renowned for its low heat input and high kinetic energy, slight surface oxidation inevitably occurred at the particle boundaries during the high-velocity impact and subsequent rapid quenching of the semi-molten particles on the substrate. Following the severe plastic deformation and mutual impingement of the particles during stacking, these thin oxide layers evolved into an intricate three-dimensional network of oxygen-rich inter-splat boundaries within the coatings [28]. Although this microscopic boundary network constitutes a minor macroscopic volume fraction, the non-magnetic oxide interlayer, severe lattice distortion, and local stress concentration combined to form a strong domain wall pinning network, which severely hindered the domain inversion [29].

**Figure 4:**
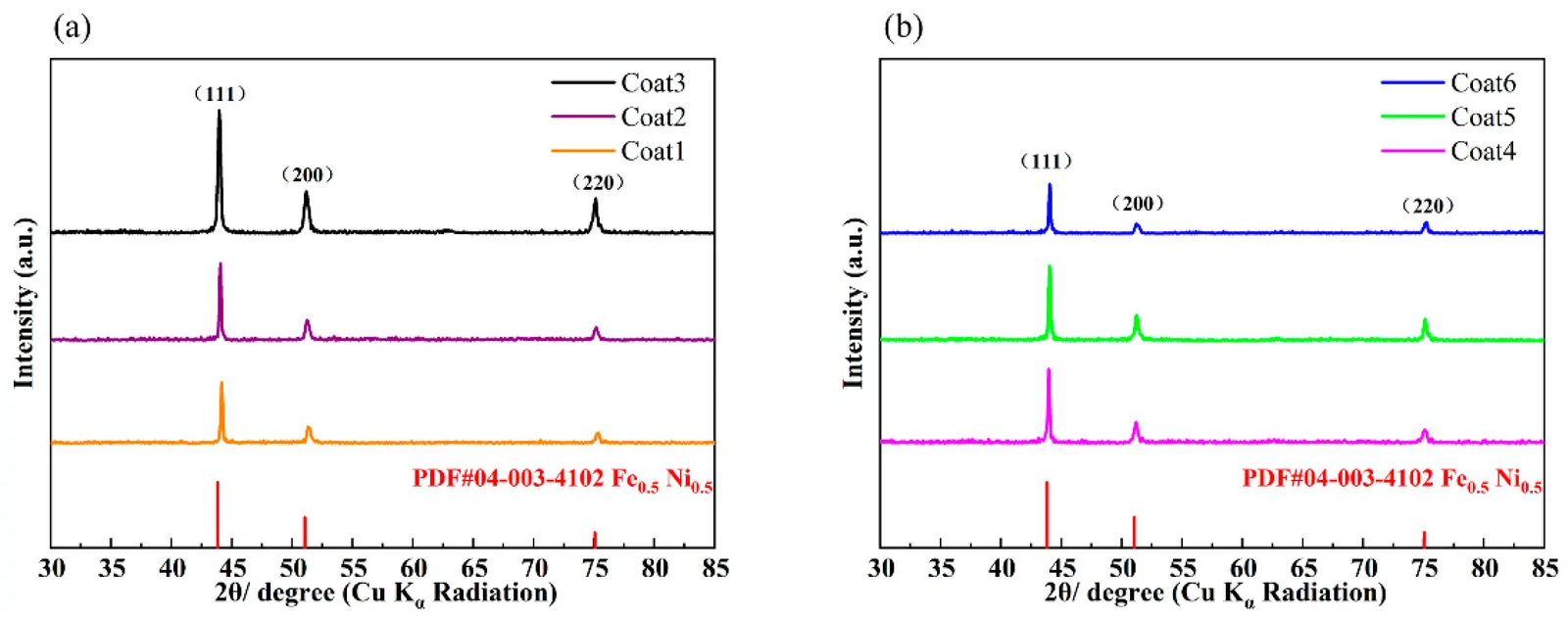
XRD patterns of the 1J38 coatings deposited on various substrates with varying thicknesses: (**a**) 0.3 mm-thick coatings (Coat1, Coat2, Coat3); (**b**) 0.5 mm-thick coatings (Coat4, Coat5, Coat6).

Crystallographic characterization of the coating surfaces across the six sample groups was performed utilizing XRD [30], with the results illustrated in Figure 4. Irrespective of the in-situ thermal accumulation induced by varying deposition thicknesses or the distinct thermophysical conduction properties of the metallic substrates, the XRD patterns of all coatings exhibited remarkable consistency. The well-defined and sharp diffraction peaks in the patterns are predominantly located at 2*θ* ≈ 43.6°, 50.8°, and 74.7°, corresponding to the principal (111), (200), and (220) crystallographic planes of the face-centered cubic (fcc) solid solution phase, respectively. These diffraction characteristics are in excellent agreement with the standard JCPDS card PDF#04-003-4102 for the Fe_0.5_Ni_0.5_ phase.

Furthermore, the baselines of the XRD patterns remain flat, with no detectable diffraction peaks corresponding to macroscopic iron/nickel oxides (e.g., Fe_2_O_3_, Fe_3_O_4_, or NiO). This pivotal crystallographic result compellingly demonstrates that the exceptionally short in-flight thermal exposure time characteristic of the HVOF process successfully shielded the core soft-magnetic atoms (Fe, Ni) within the 1J38 alloy from catastrophic deep oxidation and disruptive phase transformations. The intact preservation of the primary Fe_0.5_Ni_0.5_ face-centered cubic lattice crystallographically endows the coatings with the intrinsic potential to retain an exceptionally high saturation magnetization (*M*_s_) [31].

### 3.3. Signal Masking Effect of Ferromagnetic Substrates

Typically, the absolute effective mass of thermally sprayed soft-magnetic thin coatings (with thicknesses on the order of 0.3–0.5 mm) is exceedingly small, amounting to merely a few milligrams. During global spatial-field measurements utilizing a conventional vibrating sample magnetometer (VSM), the total magnetic response of the composite specimen (coating + substrate) is highly susceptible to being distorted, or even completely swamped, by the macroscopic background signal of the bulky substrate [32].

**Figure 5:**
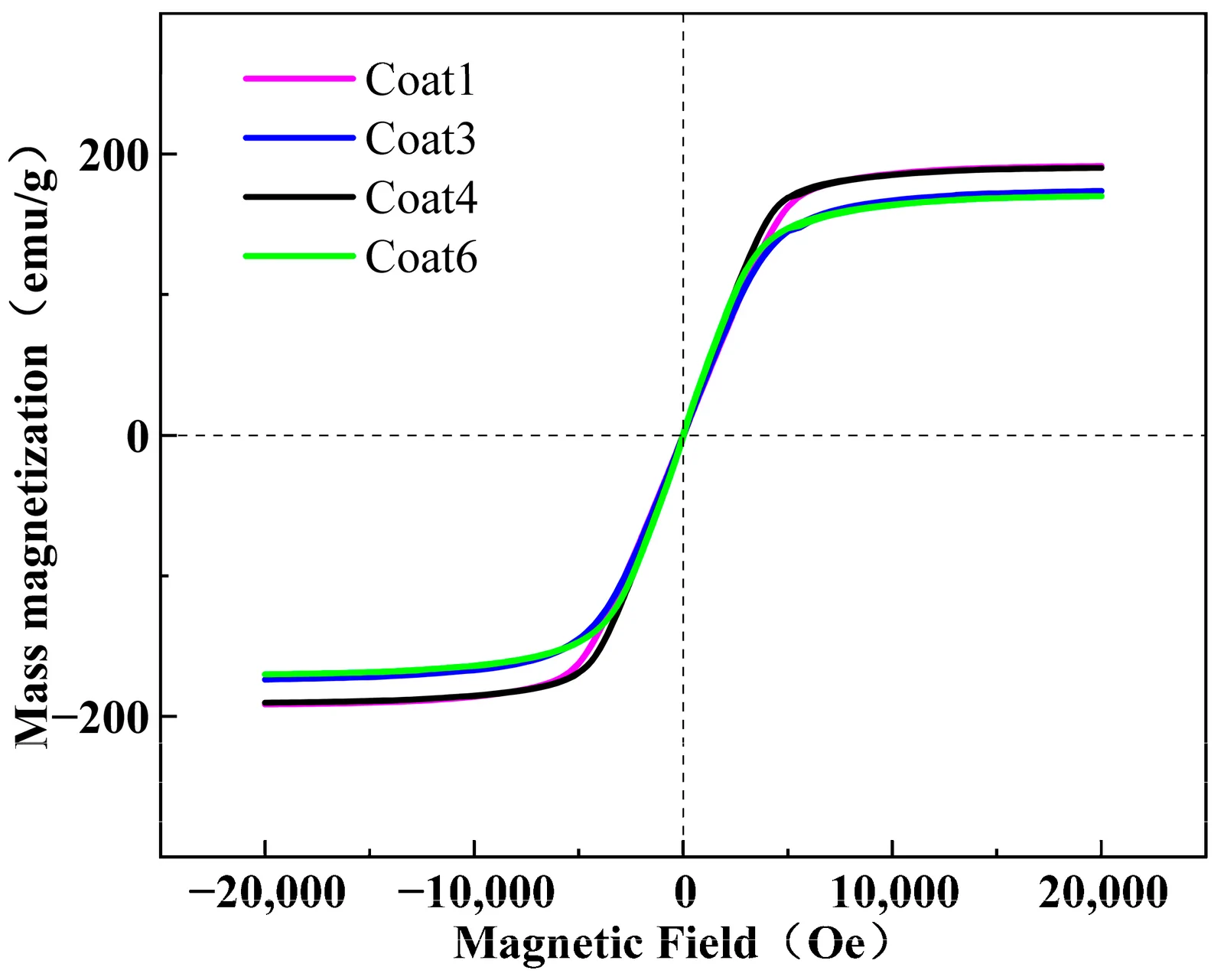
M-H hysteresis loops of the composite samples deposited on 42CrMo and GCr15 substrates.

To intuitively elucidate and circumvent this critical measurement pitfall, room-temperature magnetic response tests were initially performed on four sets of composite samples deposited on typical ferromagnetic structural steel (42CrMo) and bearing steel (GCr15) substrates (namely, the 0.3 mm-thick Coat1 and Coat3, and the 0.5 mm-thick Coat4 and Coat6). As illustrated in Figure 5, the M-H hysteresis loops of these four composite samples exhibited a remarkably high degree of spatial overlap, displaying anomalously high saturation magnetizations (with *M*_s_ values all approaching 200 emu/g).

In conjunction with the aforementioned PPMS measurements, the theoretical intrinsic *M*_s_ of the pure 1J38 soft magnetic alloy powder is merely 28.44 emu/g. Evidently, the colossal apparent magnetic moment observed in Figure 5 drastically exceeds the physical limits of the 1J38 material itself. This stark contrast demonstrates that the robust magnetic signal approaching 200 emu/g in the composite curves essentially originates in its entirety from the underlying bulky, highly ferromagnetic steel substrates. Consequently, within the measurement space, the ferromagnetic substrates exert an overwhelming signal masking effect.

Furthermore, the coercivity characteristics and initial magnetic susceptibility exhibited by these four composite curves are merely macroscopic reflections of the bulk magnetic properties inherent to the substrates themselves, thoroughly concealing the domain pinning mechanisms induced by the coating thickness effect and microstructural defects (e.g., residual stresses and oxygen-rich boundaries). It should be stressed that while the structural steel substrates dominate the global magnetic response in these measurements, the deposition of the 1J38 coating remains functionally irreplaceable. The 1J38 alloy is a dedicated thermosensitive soft magnetic material whose magnetic permeability varies sharply with temperature, providing the essential physical basis for in-situ temperature monitoring. In contrast, conventional structural and bearing steels (42CrMo and GCr15) serve strictly as mechanical load carriers and exhibit negligible permeability variation across the service temperature range (0~150 °C), making the substrate alone incapable of temperature perception. Therefore, to accurately capture the intrinsic magnetic properties of the 1J38 coatings without substrate interference, it is imperative to employ pure Cu substrates featuring an exceptionally weak magnetic background, complemented by a rigorous mathematical decoupling algorithm for in-depth signal stripping.

### 3.4. Intrinsic Magnetic Properties and Coercivity Degradation Mechanisms of the Coatings

**Figure 6:**
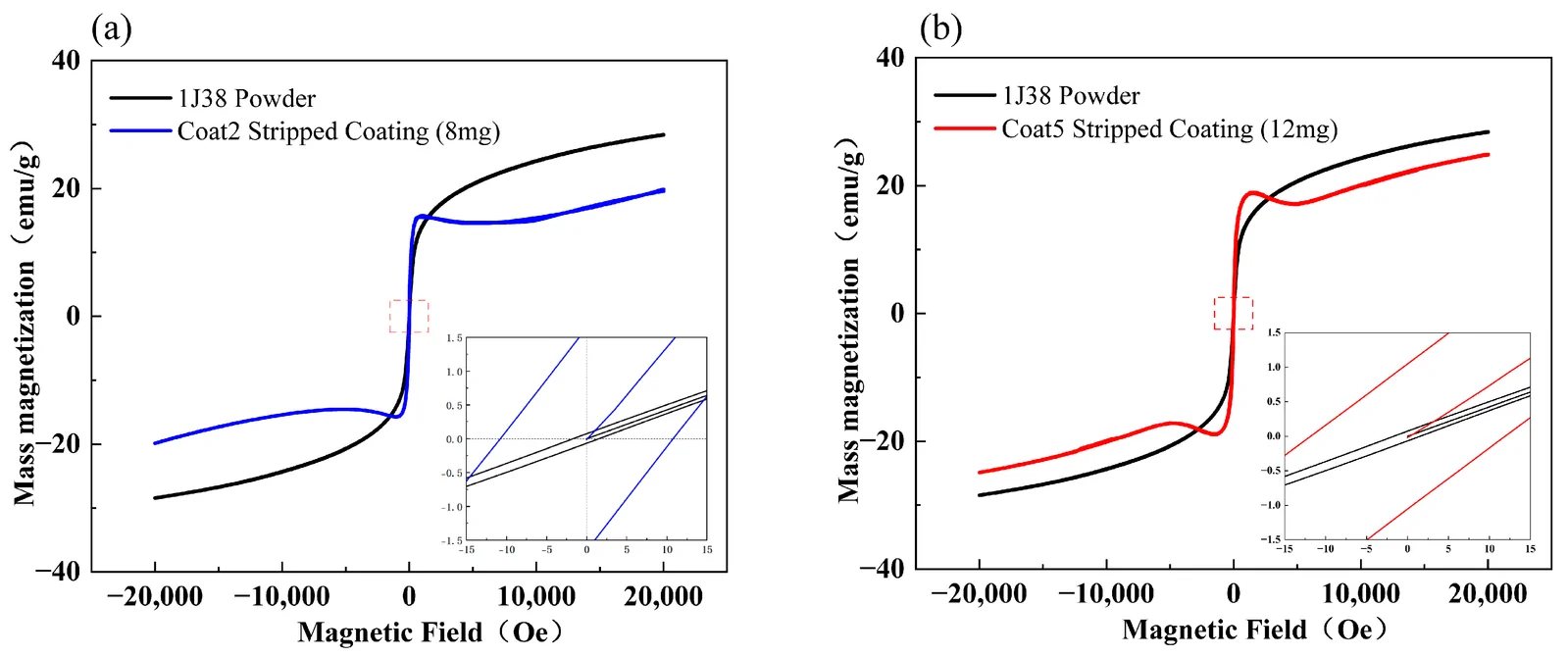
Comparison of the intrinsic hysteresis loops of the 1J38 coatings (after Cu substrate background subtraction) and the pristine alloy powder: (**a**) 0.3 mm-thick coating (Coat2, blue line); (**b**) 0.5 mm-thick coating (Coat5, red line). The black curves in both panels represent the raw 1J38 powder. The insets display the magnified views of the low-field region indicated by the red dashed boxes, illustrating the coercivity (*H*_c_) behavior near zero magnetic field.

To circumvent the severe signal masking effect induced by the ferromagnetic substrates, this study employed the weakly magnetic pure Cu plate as a reference model substrate in combination with a precise data decoupling algorithm to perform background signal stripping on the raw data of Coat2 (Cu substrate, 0.3 mm) and Coat5 (Cu substrate, 0.5 mm). Consequently, the high-fidelity intrinsic hysteresis loops of the coatings were successfully reconstructed, as depicted in Figure 6.

The decoupled results reveal two salient macroscopic physical features: first, regarding the saturation magnetization (*M*_s_), the raw 1J38 powder exhibits an intrinsic *M*_s_ of 28.44 emu/g under an external applied field of 20,000 Oe. Upon deposition into coatings, the *M*_s_ values of both groups undergo noticeable attenuation: the 0.3 mm-thick coating (Coat2) decreases to approximately 20.41 emu/g, whereas the 0.5 mm-thick coating (Coat5) reaches approximately 25.13 emu/g, outperforming the thinner coating yet remaining inferior to the raw powder. The overall degradation of the intrinsic magnetic moment in the coatings is primarily attributed to the slight boundary oxidation and severe lattice distortion accompanying the rapid quenching process, which result in a relative reduction in the effective soft-magnetic core volume. Conversely, the relative recovery of *M*_s_ observed in the 0.5 mm-thick coating (reaching ~25.13 emu/g) is predominantly governed by thermal accumulation during successive spray passes. For the thinner coating (0.3 mm), molten droplets directly contact the high-heat-dissipation cold substrate, experiencing ultra-fast quenching (>10^7^ K/s) that freezes substantial short-range atomic disorder and lattice distortion into the splats. In contrast, for the thicker coating (0.5 mm), the thermal build-up from multi-pass deposition moderately mitigates the instantaneous cooling rate during splat solidification, thereby promoting localized atomic ordering and restoring the crystallographic exchange coupling within the unoxidized splat cores [33].

Second, in the low-field region (±2000 Oe), the magnetization trajectories of the Coat2 and Coat5 coatings climb steeply, with their apparent magnetizations rapidly surpassing that of the raw powder. This phenomenon stems from the drastic alteration in the macroscopic shape anisotropy of the material: the raw powder possesses a large demagnetization factor (*N*_d_ ≈ 1/3) due to its perfect spherical morphology, causing its initial magnetization curve to tilt severely under the hindrance of a strong demagnetizing field. Conversely, the interior of the HVOF coatings exhibits a two-dimensional flattened lamellar stacking (*N*_d_ ≈ 0), which completely eliminates the constraints imposed by the demagnetizing field. Meanwhile, the decoupled intrinsic hysteresis loops of the coatings display an exceptionally unique wasp-waist distortion in the mid-to-low field range (around ±5000 Oe). Combined with microstructural characterizations, this anomalous macroscopic distortion originates from an asynchronous magnetization reversal mechanism induced by microstructural heterogeneity. The unoxidized 1J38 splat cores possess low anisotropy and preferentially undergo rapid domain wall displacement under low external fields. In contrast, the inter-splat boundaries, which are enriched with non-magnetic oxide films and subjected to intense localized residual stress concentrations, act as high-barrier pinning networks with high switching fields, lagging substantially during field reversal. The superposition of these two distinct switching field distributions produces the constricted distortion observed near the zero-crossing points of the hysteresis loops [34].

Finally, the magnified low-field region (inset) in Figure 6 distinctly delineates the coercivity (*H*_c_) comparison between the coatings and the raw powder. The pristine, stress-free powder exhibits an *H*_c_ of merely 1.6 Oe, whereas the *H*_c_ of the 0.3 mm-thick coating (Coat2) deteriorates to 10.8 Oe; as the deposition thickness further increases to 0.5 mm (Coat5), the *H*_c_ continues to escalate to 11.9 Oe. This significant degradation relative to the raw powder indicates that intra-coating growth stress gradients induced by thickness accumulation play a dominant role in performance evolution. Although inter-pass thermal accumulation slightly moderates splat-level atomic disorder, the overall deposition temperature during HVOF (typically below 250 °C) is far insufficient to activate macroscopic stress-relief or recrystallization mechanisms in 1J38 Permalloy (which typically require temperatures exceeding 600 °C). Consequently, as the coating thickness escalates from 0.3 mm to 0.5 mm, the repeated supersonic peening impacts, micro-quenching tensile stresses, and through-thickness thermal contraction gradients continuously pile up cumulative intra-coating growth stresses (*σ*_growth_) and high-density dislocation networks within the bulk deposit. Because the bulk coating thickness (300–500 μm) far exceeds the localized interfacial boundary layer, the macroscopic magnetic response is overwhelmingly governed by these internal growth stresses rather than substrate-specific interfacial thermal mismatch stresses. Through strong magnetoelastic coupling, these cumulative intra-coating stress fields comprehensively amplify the pinning force exerted by the inter-splat boundary defect networks, dramatically elevating the magnetic moment reversal barrier of the entire system and ultimately governing the progressive deterioration of coercivity in the thicker coatings [35].

## 4. Discussion

### 4.1. Intrinsic Magnetic Properties and Underlying Mechanisms of Degradation in the Coatings

The microstructural evolution and macroscopic magnetization dynamics exhibited by the 1J38 soft-magnetic coatings prepared via the HVOF spraying process are jointly governed by extreme non-equilibrium thermodynamic processes and multiphase defect networks. First, the coatings demonstrate exceptional attributes regarding the retention of their saturation magnetization (*M*_s_) potential. During supersonic impact and rapid quenching, the peripheries of the semi-molten particles undergo slight oxidation, evolving into intricate three-dimensional inter-splat boundary networks within the coatings. Nevertheless, XRD analysis confirms that the coatings do not undergo massive phase transformations, faithfully preserving the face-centered cubic (FCC) lattice structure of the pristine alloy. Consequently, the as-sprayed coatings maintain their *M*_s_ at the same high order of magnitude as the raw powder, averting the catastrophic drop in magnetic moment typically induced by severe oxidative depletion. Furthermore, the moderate thermal accumulation during multi-pass deposition slightly mitigates the extreme quenching rate during splat solidification, preserving atomic-scale ferromagnetic exchange coupling and allowing *M*_s_ to recover in thicker deposits, which crystallographically validates the highly effective preservation of the material’s core soft-magnetic potential by this spraying process.

Second, following the subtraction of the substrate signal, the intrinsic hysteresis loops reveal a profound reshaping of the coating’s shape anisotropy: the two-dimensional flattened lamellar stacking eradicates the severe demagnetizing field constraints inherent to the spherical powder, enabling the magnetization trajectory of the coating to ascend steeply in the low-field regime. Concurrently, the loops exhibit a distinctive wasp-waist distortion within the mid-to-low field range. This anomalous feature originates from an asynchronous magnetization reversal competition between regions with contrasting switching barriers. The unoxidized splat cores, exhibiting low magnetocrystalline anisotropy and high structural order, undergo rapid domain wall displacement under low external fields [36]. Conversely, the inter-splat boundary networks do not represent an intrinsic hard ferromagnetic phase, but rather a high-barrier pinning network: the non-magnetic/antiferromagnetic oxide films interrupt ferromagnetic exchange coupling, while severe localized plastic strains induce strong magnetoelastic anisotropy [37,38]. Consequently, these boundary networks require much higher external fields to overcome domain pinning and rotate moments. The temporal decoupling between the fast-switching soft splat cores and the lagging high-barrier boundary networks superimposes, ultimately manifesting as the macroscopic wasp-waisted constriction.

Finally, regarding the coercivity (*H*_c_) comparison, the pristine stress-free powder exhibits an *H*_c_ of merely 1.6 Oe, whereas upon deposition, the *H*_c_ values escalate to 10.8 Oe for the 0.3 mm-thick coating and 11.9 Oe for the 0.5 mm-thick coating. When interpreting the stress-induced magnetic degradation, two distinct residual stress contributions must be physically distinguished: interfacial thermal mismatch stress (*σ*_th_) and intra-coating growth stress (*σ*_growth_). The thermal mismatch stress arises from the disparity in thermal expansion coefficients (*α*_1J38_ ≈ 10.8 × 10^−6^ K^−1^, *α*_steel_ ≈ 11.8~12.2 × 10^−6^ K^−1^ and *α*_Cu_ ≈ 16.8 × 10^−6^ K^−1^). While the larger thermal contraction of the Cu substrate generates a higher compressive *σ*_th_ in the immediate vicinity of the interface, interfacial stress fields decay rapidly away from the boundary according to classical shear-lag models [39]. For the relatively thick coatings investigated in this work (300~500 μm), the macroscopic magnetic response of the bulk coating volume is primarily governed by intra-coating growth stresses (*σ*_growth_), which originate from the cumulative superposition of micro-quenching stresses and supersonic particle peening stresses across multi-pass spray runs. Because the in-flight particle kinetics and lamellar stacking mechanisms are substrate-independent, the observed escalation of *H*_c_ with increasing thickness faithfully mirrors the intrinsic accumulation of intra-splat stress fields and dislocation networks.

Importantly, the slight recovery of saturation magnetization (*M*_s_) and the concurrent degradation of coercivity (*H*_c_) in thicker coatings operate on two distinct physical scales and do not represent a mechanistic contradiction. While *M*_s_ is an intrinsic property reflecting atomic-scale exchange interactions—benefiting from moderated splat quenching rates via inter-pass thermal accumulation during solidification—*H*_c_ is an extrinsic, structure-sensitive parameter governed by mesoscopic defect pinning [40]. Because the macroscopic deposition temperature (<250 °C) remains well below the thermal threshold for dislocation recovery or stress relaxation in Fe-Ni alloys (>600 °C), continuous multi-pass peening and thermal contraction gradients inevitably amplify the bulk intra-coating growth stress fields (*σ*_growth_). Through intense magnetoelastic coupling, these unrelaxed internal stress fields comprehensively amplify the pinning force exerted by the inter-splat defect boundary networks, elevating the energy barrier for domain wall displacement and dictating the progressive elevation of *H*_c_. The most pivotal conclusion, however, lies herein: despite enduring the extreme deposition process, the intrinsic coercivity of the coatings is successfully constrained within a superior soft-magnetic regime of approximately 10–12 Oe. Such a marginal deterioration in magnetic performance remains at an exceptionally low level that is thoroughly acceptable for engineering applications, compellingly validating the feasibility of employing the HVOF process to fabricate high-performance Fe-Ni-Cr soft-magnetic coatings on structural components.

### 4.2. Theoretical Insights and Engineering Application Value

From a theoretical and methodological perspective, this study systematically evaluates the profound sources of error encountered in the conventional magnetic characterization of ultra-thin soft-magnetic coatings. Ferromagnetic substrates (e.g., 42CrMo or GCr15) exert an overwhelming “signal masking” effect, yielding an artifactual, apparent saturation magnetization of up to approximately 200 emu/g that originates from the steel substrate rather than a genuine physical enhancement of the 1J38 deposit (whose intrinsic *M*_s_ is merely 28.44 emu/g). Mistaking this background-dominated signal for an enhanced coating property leads to severe data misinterpretation, thoroughly obscuring the true physical behavior of the coatings. By introducing weakly magnetic pure Cu substrates in conjunction with a substrate signal stripping algorithm, this study successfully eradicates the signal-to-noise distortion induced by the composite background and precisely reconstructs the intrinsic hysteresis loops, thereby establishing a rigorous methodological paradigm for the high-precision characterization of soft-magnetic films/coatings.

From an engineering application perspective, this study effectively overcomes the physical bottlenecks of traditional bulk 1J38 alloys, namely their susceptibility to mechanical stress, high density, and difficulty in conformal processing. Employing the HVOF surface engineering technique not only circumvents the performance degradation induced by subtractive machining, but also provides an innovative solution for the intelligent condition monitoring of high-end equipment. Specifically regarding the in-situ early warning of thermally induced bearing failures within enclosed and complex oil–gas environments, this Fe-Ni-Cr alloy coating can be non-destructively and conformally deposited onto the surfaces of critical moving components, demonstrating immense engineering application potential and cutting-edge significance by leveraging its highly sensitive permeability–temperature coupling properties in conjunction with eddy current sensors.

### 4.3. Research Limitations and Future Perspectives

While this study successfully elucidates the intrinsic magnetic properties and degradation mechanisms of the HVOF coatings, certain limitations inevitably remain. First, the dual-phase competition model between the “soft-magnetic cores” and the “hard-magnetic network” is primarily deduced from macroscopic hysteresis loop distortions and static EDS elemental mapping, currently lacking direct in-situ observational evidence of the dynamic domain wall pinning processes under alternating external fields. Second, the degradation mechanism driven by residual stress on coercivity is only qualitatively evaluated across two macroscopic thickness gradients, falling short of establishing a precise quantitative mapping between the three-dimensional stress gradients and the magnetoelastic coupling energy. Furthermore, the current characterizations are predominantly confined to room temperature and quasi-static alternating magnetic fields. Additionally, direct magnetic validation on isolated freestanding coatings was not pursued because chemical substrate dissolution or mechanical sectioning can introduce unintended chemical alterations or parasitic machining stresses into strain-sensitive 1J38 deposits. Under these circumstances, paired non-destructive background subtraction using high-purity Cu model substrates provides an effective and non-invasive approach to capture the undisturbed as-deposited magnetic response.

Building upon these limitations, future research could be advanced along the following dimensions: first, employing in-situ micromagnetic characterization techniques, including Magnetic Force Microscopy (MFM) or Lorentz Transmission Electron Microscopy (LTEM), to directly elucidate the dynamic modulation effects of oxygen-rich boundaries and residual stresses on magnetic domain motion. Second, to address the practical engineering requirements of bearing temperature sensors, it is imperative to evaluate the service reliability of the coatings across a broad temperature range and under high-frequency alternating magnetic fields. Third, exploring the implementation of post-deposition annealing treatments (e.g., in a vacuum or reducing atmosphere) is recommended to effectively relieve residual stresses and mitigate boundary oxidation, thereby fundamentally reversing the magnetic degradation trend in thicker coatings and accelerating their industrial application in modern electronic devices.

## 5. Conclusions

This study systematically investigated the effects of the high-velocity oxygen-fuel (HVOF) spraying process on the microstructural evolution and macroscopic magnetization dynamics of 1J38 soft-magnetic alloy coatings. The results demonstrate that while the ultra-fast thermal cycling of the HVOF process faithfully preserves the principal face-centered cubic (FCC) phase of the alloy powder, the severe lamellar stacking deformation and slight oxidation at the particle peripheries inevitably introduce an oxygen-rich inter-splat boundary network. To counteract the catastrophic signal masking artifacts induced by ferromagnetic steel substrates (42CrMo and GCr15) during conventional VSM characterizations, this study employed a weakly magnetic pure Cu substrate in conjunction with a mathematical decoupling algorithm, successfully extracting the high-fidelity intrinsic hysteresis loops of the coatings. The intrinsic data not only corroborate the elimination of low-field shape anisotropy by the two-dimensional flattened coatings but also distinctly reveal a unique wasp-waist distortion in the mid-to-low field regime, which originates from the asynchronous magnetization reversal between the easily reversed, unoxidized soft-magnetic splat cores and the strongly pinned, high-barrier inter-splat boundary networks (combining boundary oxide films and localized stress concentrations) under alternating magnetic fields. Furthermore, the thickness accumulation (from 0.3 mm to 0.5 mm) induces a drastic surge in macroscopic residual stresses, which significantly amplifies the resistance against domain wall motion via the magnetoelastic coupling effect, ultimately governing the progressive degradation of coercivity in the as-sprayed thicker coating.

## Figures and Tables

**Table 1:** Chemical composition of the 1J38 alloy powder.

Element	Fe	Ni	Cr	Mn	Si	C	S	P
mass fraction/%	Balance	37.82	12.92	0.46	0.25	0.04	0.02	0.02

**Table 2:** Summary of the chemical compositions and macroscopic magnetic parameters of the substrate materials.

Substrate Material	Main Chemical Composition (wt.%)	Magnetic Property	Typical Saturation Magnetization *M*_s_ (emu/g)	Typical Coercivity *H*_c_ (Oe)
42CrMo Steel	Fe (Bal.), C (0.38–0.45), Cr (0.9–1.2), Mo (0.15–0.25)	Typical ferromagnetic	~200	15–30
Pure Cu Plate	Cu (≥99.9)	Diamagnetic	≈0	≈0
GCr15 Steel	Fe (Bal.), C (0.95–1.05), Cr (1.4–1.65)	Strong ferromagnetic	~200	40–60

**Table 3:** Process parameters of the high-velocity oxygen-fuel (HVOF) spraying.

Propane Flow Rate	Oxygen Flow Rate	Traverse Speed	Powder Feed Rate	Spray Distance
(L/min)	(L/min)	(mm/s)	(g/min)	(mm)
38	88	350	40	180

**Table 4:** Experimental sample groups.

Experiment Number	Base Material	Coating Thickness
Coat1	42CrMo	0.3 mm
Coat2	Cu	0.3 mm
Coat3	GCr15	0.3 mm
Coat4	42CrMo	0.5 mm
Coat5	Cu	0.5 mm
Coat6	GCr15	0.5 mm

## Data Availability

The original contributions presented in this study are included in the article. Further inquiries can be directed to the corresponding author.
